# Giant Fibrovascular Polyp of the Esophagus: A Novel Technical Approach

**DOI:** 10.1155/2012/562363

**Published:** 2012-06-25

**Authors:** Juan José Trakál, Guillermo Jorge Sarquis, Juan Antonio Muñoz, Sergio Barril, Esteban Trakál, Lucas Armando, Rosa Carolina Gorordo Ipiña

**Affiliations:** ^1^Gastroenterology Service, Clínica Universitaria Reina Fabiola, Oncativo 1248 St., Córdoba 5000, Argentina; ^2^Thorax Surgery Service, Clínica Universitaria Reina Fabiola, Oncativo 1248 St., Córdoba 5000, Argentina; ^3^Histopathology Service, Clínica Universitaria Reina Fabiola, Oncativo 1248 St., Córdoba 5000, Argentina

## Abstract

Fibrovascular polyps (FVPs) of the esophagus are rare, and their course is usually indolent until reaching enormous proportions. It is a dramatic entity owing to its tendency to cause bizarre complications. We describe a 49-year-old female patient with sudden dyspnoea that required digital maneuvers to clear the airway. After diagnosing, an FVP of the esophagus, a video-assisted endocavitary surgery was made. Histopathological examination revealed a fibrovascular polyp. Endoscopic controls after excision show no mass or symptoms recurrence.

## 1. Introduction

Fibrovascular polyps (FVPs) of the esophagus are benign, rare, intraluminal tumors [1–8] and represent less than 2% of esophageal tumors [1–3, 5–7]. The most common complaints include dysphagia, foreign body sensation, weight loss, regurgitation of the mass, sudden death, and asphyxiation when the polyp regurgitates and occludes the larynx [1–9].

## 2. Case Report

A 49-year-old female patient described an episode of sudden dyspnoea that required digital maneuvers to clear the airway. She described that during the maneuver a smooth mass was detached from the pharynx and swallowed. Similar episodes were repeated in two occasions. During examination a progressive dysphagia to solids was noted. No history of loss weight, cough, or hematemesis was noted. She had no comorbidity of interest.

A CT scan was performed revealing a soft tissue mass in the esophagus, extending from the level of the cervical esophagus to the lower esophagus with no clear relation to the esophagus wall. 

**Figure 1:**
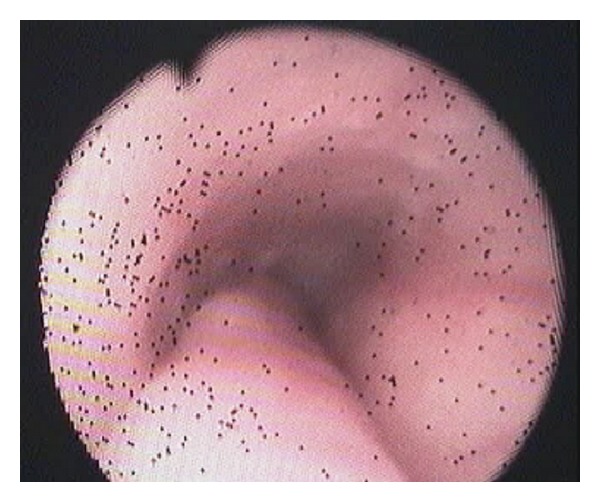
Sausage-shaped mass obstructing the esophageal lumen.

The upper endoscopy showed a sausage-shaped mass obstructing the esophageal lumen, arising from the upper esophageal sphincter and ending 13 cm below (Figure 1).

**Technical Approach**

**Figure 2:**
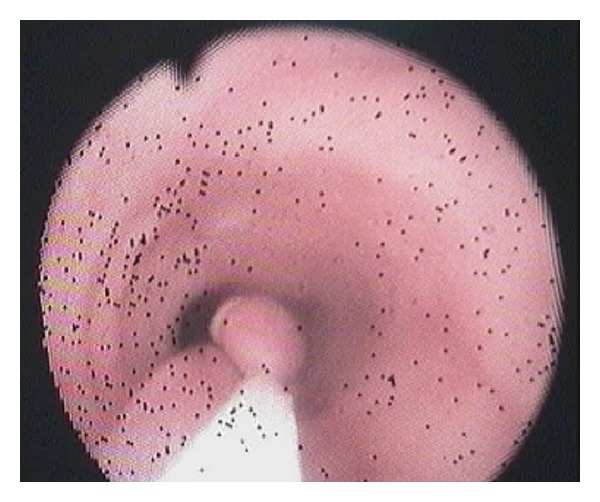
Sneared mass from his distal portion.

**Figure 3:**
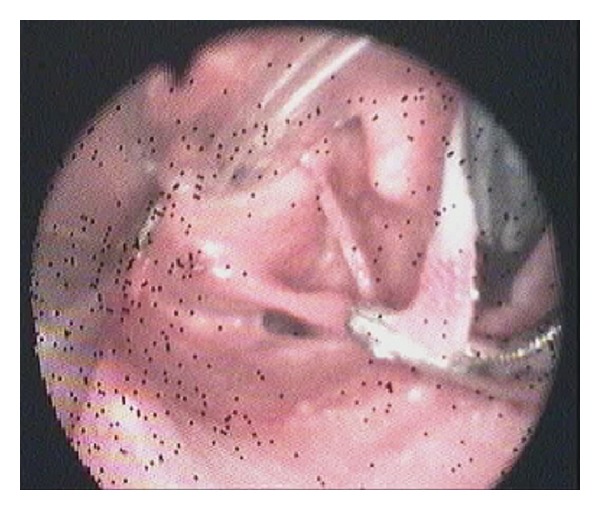
Base of the tumor electrocoagulated.

**Figure 4:**
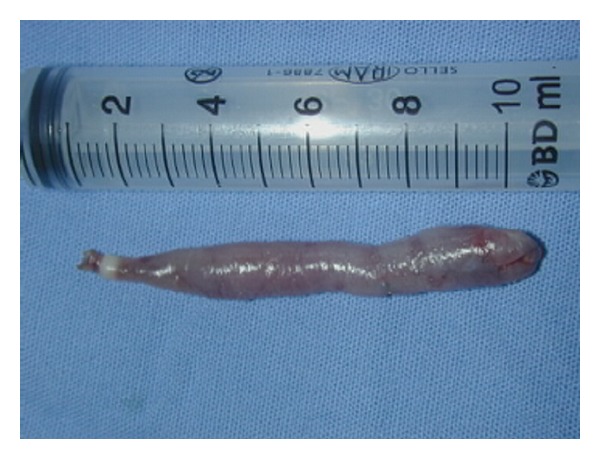
Sausage shaped mass after sugery.

Tracheal intubation previous to upper endoscopy was made; the mass measured 13 × 1,5 cm and was sneared from his distal portion (Figure 2) and pulled into the mouth giving a clear image of the implantation base at the Killian's triangle. Once the distal portion of the tumor was fixed into the mouth and the base trapped by means of Kantrowitz forceps, the video-assisted endocavitary surgery started. The tumor was pulledby means of Magill forceps, and using laparoscopic surgical instrumentation and video assistance during the procedure the base of the tumor was electrocoagulated with the hook right above the Kantrowitz forceps (Figure 3) providing a clear control of possible hemorrhage (Figure 4). Immediate endoscopy was performed looking for possible complications of the procedure with negative results.

Twenty-four hours later the patient was able to eat soft meals and 48th  hours after the procedure she was discharged.

**Figure 5:**
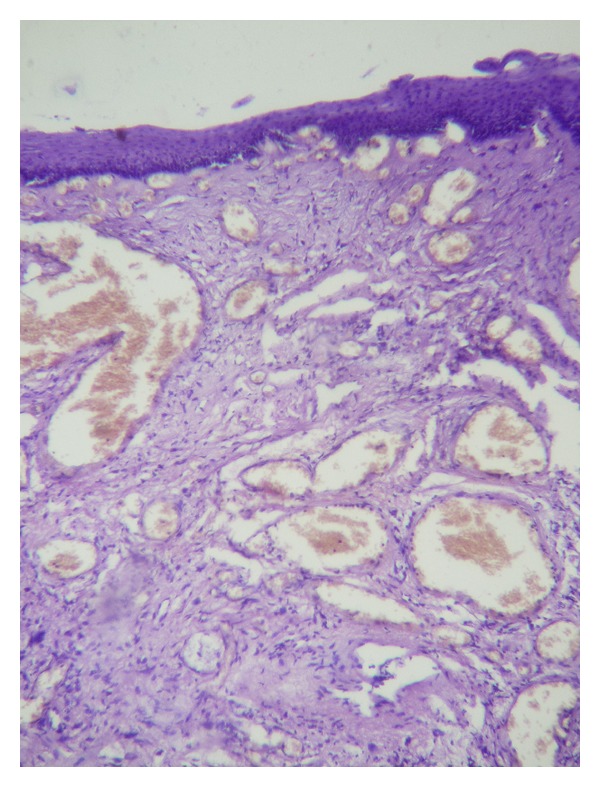
Polypoid lesion covered by squamous epithelium with acanthosis lining edematous stroma with areas of fibrosis. Blood vessels and abundant lymphoplasmacytic infiltrate.

Histopathological examination revealed a polypoid lesioncoveredbysquamous epithelium withacanthosis liningedematousstromawith areas offibrosis.Blood vessels andabundantlymphoplasmacytic infiltrate (Figure 5), concluding fibrovascular polyp.

Endoscopic controls were made 4 months and 24 months after excision with no mass or symptoms recurrence.

## 3. Discussion

Despite its benign condition, it has a life threatening feared complication. This is an unusual tumor and different approaches have being proposed, including transverse cervical incision, transoral resection under direct visualization, endoscopic ligation and electrocoagulation of the pedicle (small size FVP), biapproach surgical technique (eoesophagostomy plus gastrostomy) and CO_2_ laser under laryngoscope [1–9].

The novel technical approach that we propose includes the safety and accuracy of transverse cervical incision, the simplicity of an endoscopic procedure, scarless, with early discharge and recovery.

It is important to consider that if FVP has become giant, it means we have got late to the patient, and this may be either because the patient shows no complaints, or because its symptoms have not been studied enough due to their nonspecificity.
